# Enhanced breast cancer therapy with nsPEFs and low concentrations of gemcitabine

**DOI:** 10.1186/s12935-014-0098-4

**Published:** 2014-10-12

**Authors:** Shan Wu, Jinsong Guo, Wendong Wei, Jue Zhang, Jing Fang, Stephen J Beebe

**Affiliations:** College of Engineering, Peking University, Beijing, 100871 China; Academy for Advanced Interdisciplinary Studies, Peking University, Beijing, 100871 China; Frank Reidy Research Center for Bioelectrics, Old Dominion University, 4211 Monarch Way, Suite 300, Norfolk, VA 23508 USA

**Keywords:** Breast cancer, MCF-7, MDA-MB-231, Nanosecond pulsed electric fields (nsPEFs), Non-thermal, Gemcitabine, Synergism, Clonogenics, Apoptosis, Necrosis (necroptosis)

## Abstract

**Background:**

Chemotherapy either before or after surgery is a common breast cancer treatment. Long-term, high dose treatments with chemotherapeutic drugs often result in undesirable side effects, frequent recurrences and resistances to therapy.

**Methods:**

The anti-cancer drug, gemcitabine (GEM) was used in combination with pulse power technology with nanosecond pulsed electric fields (nsPEFs) for treatment of human breast cancer cells *in vitro*. Two strategies include sensitizing mammary tumor cells with GEM before nsPEF treatment or sensitizing cells with nsPEFs before GEM treatment. Breast cancer cell lines MCF-7 and MDA-MB-231 were treated with 250 65 ns-duration pulses and electric fields of 15, 20 or 25 kV/cm before or after treatment with 0.38 μM GEM.

**Results:**

Both cell lines exhibited robust synergism for loss of cell viability 24 h and 48 h after treatment; treatment with GEM before nsPEFs was the preferred order. In clonogenic assays, only MDA-MB-231 cells showed synergism; again GEM before nsPEFs was the preferred order. In apoptosis/necrosis assays with Annexin-V-FITC/propidium iodide 2 h after treatment, both cell lines exhibited apoptosis as a major cell death mechanism, but only MDA-MB-231 cells exhibited modest synergism. However, unlike viability assays, nsPEF treatment before GEM was preferred. MDA-MB-231 cells exhibited much greater levels of necrosis then in MCF-7 cells, which were very low. Synergy was robust and greater when nsPEF treatment was before GEM.

**Conclusions:**

Combination treatments with low GEM concentrations and modest nsPEFs provide enhanced cytotoxicity in two breast cancer cell lines. The treatment order is flexible, although long-term survival and short-term cell death analyses indicated different treatment order preferences. Based on synergism, apoptosis mechanisms for both agents were more similar in MCF-7 than in MDA-MB-231 cells. In contrast, necrosis mechanisms for the two agents were distinctly different in MDA-MB-231, but too low to reliably evaluate in MCF-7 cells. While disease mechanisms in the two cell lines are different based on the differential synergistic response to treatments, combination treatment with GEM and nsPEFs should provide an advantageous therapy for breast cancer ablation *in vivo*.

## Background

Breast cancer is the most common malignancy in women that affected an estimated three million women worldwide in 2008 alone and is a major cause of morbidity and mortality [1]. Breast cancer growth rates vary considerably among cancer patients; they grow significantly faster in younger women [2-7], in recurrent cancers [2] and in patients with BRCA1 and BRCA2 mutations. [4]. Incidences of ductal carcinoma *in situ* (DCIS) have increased 5-fold over the last 30 years (5.8-32.5/100000). Although the incidence of invasive breast cancer has increased much less, it is much more common than DCIS (124.3/100000) [7].

While surgical approaches are frequent, chemotherapy is a major approach in treating breast cancer, often with anthracyclines and taxanes. However, there are significant toxicities with both drugs [8]. In locally advanced and metastatic breast cancers, gemcitabine (GEM) has been used in anthracycline-resistant breast cancer in combination with paclitaxel [9] or cisplatin [10,11]. In HER2-positive metastatic disease, GEM also has been used in combination with trastuzumab, a monoclonal antibody that inhibits HER2/neu (ErbB-2) signaling [12,13]. However, resistances to all of these agents are common. Associations between resistance and acquisition of epithelial mesenchymal transition and cancer stem cell-like phenotypes may be responsible [14]. Specifically, cisplatin or paclitaxel treated residual cells displayed higher proliferation markers and cancer stem cell markers and exhibited significantly higher tumour burden than untreated cells in a mouse xenograph model [15].

Gemcitabine (dFdC or GEM), an analog of deoxycytidine, is an anticancer nucleoside pro-drug that is phosphorylated to mono- di- and tri-phosphorylated metabolites dFdCMP, dFdCDP and dFdCTP, respectively. It is well characterized as a radiosensitizer. One likely metabolic action is inhibition of ribonucleotide reductase, leading to a depletion of dATP [16,17]. Maximum radiosensitization occurs when cells are distributed in S-phase and dATP is diminished. GEM metabolites have other effects on regulatory processes that enhance GEM actions causing a unique “self-potentiation” effect [18].

In most recent clinical trials for breast cancer, GEM was used in combinations with typical chemotherapeutic drugs and/or monoclonal antibodies to growth factors. Only a few trials have investigated unique approaches to address these diseases [19,20]. While many treatment regimens have shown some promise, treatment failures are not uncommon and drug resistances continue to be major barriers for successful treatments. Resistances against most if not all chemotherapeutic agents appear to be inevitable and many resistance mechanisms have been characterized [21-23]. For women with triple-negative breast cancer, survival time from distant recurrence to death was 9 months [24]. Clearly, unique treatment options need to be investigated beyond adding and/or deleting chemotherapeutic agents in a cocktail of drugs.

One novel pre-clinical treatment strategy uses pulse power technology, which is used in high power physics and engineering applications; it is now being developed for medical applications [25]. This unique strategy delivers electric pulses with low, non-thermal energy (mJ/cc), but instantaneous high power (GW) for ultra-short durations (nanoseconds) and high electric fields (10s of kV/cm), giving rise to nanosecond pulsed electric fields (nsPEFs). When considered in the frequency domain, these extremely short pulse durations and/or their short (fast) rise and fall times are transformed into high frequency components that have greater possibilities for permeabilizing intracellular vesicles [26] or dissipating the mitochondria membrane potential [27]. NsPEFs can also trigger other cell functions, including intracellular calcium fluctuations [28,29], phosphatidylserine translocation [30], DNA damage [31,32], unique stress responses [33] as well as activation of several different kinase signaling pathways and phosphorylation of their downstream substrates [34-36]. They have also been shown to induce apoptosis and other forms of cell death [37,38].

Pulse power using nsPEFs with repetitive pulses has been shown to eliminate mouse B16f10 melanoma [39] and was shown to induce apoptosis and other forms of cell death in B16f10 melanoma [40] and mouse Hepa1-6 hepatocellular carcinoma (HCC) [41] *in vivo*. Successful elimination of mouse and human basal cell carcinomas [42,43], squamous cell carcinoma [44] and human pancreatic carcinoma have also been reported [45]. *In vitro* studies demonstrated induction of caspase-dependent and caspase-independent cell death mechanisms through intrinsic mitochondria-mediated pathways; extrinsic apoptosis pathways were not required for nsPEF-induced cell death [38]. This shows that nsPEFs can bypass cancer mutations that evade apoptosis induction through mechanisms at either the DISC or the apoptosome, two major complexes responsible for caspase-activation and apoptosis [46].

Considering that mechanisms of action of these treatments are likely different, it is possible to achieve higher therapeutic effects with lower, non-toxic concentrations of GEM when combined with nsPEFs. We applied this novel technology of nsPEF in combination with GEM, which has been shown to eradicate MCF-7 and MDA-MB-231 cells *in vitro* [47]. Since nsPEFs have been shown to delete other cancers, because breast tumor would be readily accessible to nsPEF electrodes and because GEM has been used as a sensitizing drug, investigations were carried to determine if low, non-toxic concentrations of GEM could be used to sensitize breast cancer cells to nsPEFs or nsPEFs could be used to sensitize cells to low doses of GEM such that electric fields and/or fewer pulse numbers could be used. Results not only indicate enhanced efficacy for combinations of nsPEFs and GEM, but also reveal some insight into differences in cell death and cancer disease mechanisms for MCF-7 and MDA-MB-231 breast cancer cells in response to GEM and nsPEFs.

## Results

### Breast cancer cell sensitivity to gemcitabine

**Figure 1:**
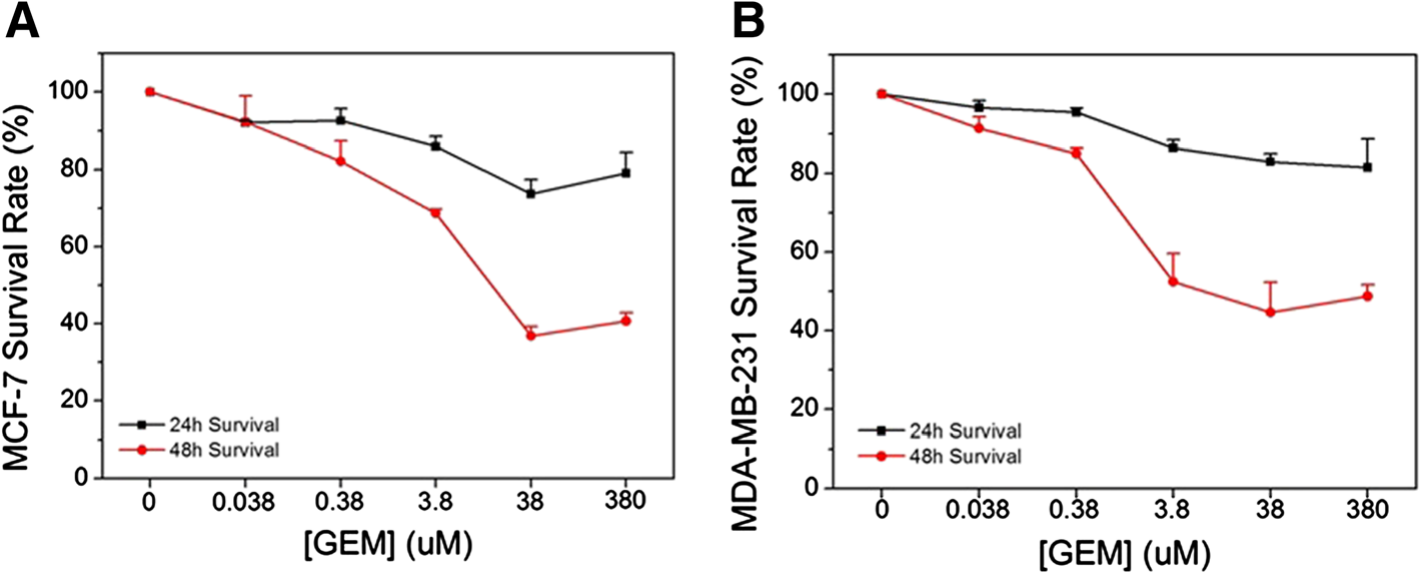
**Dose Responses for gemcitabine – The survival fractions are shown for MCF-7cells (A) and MDA-MB-231 cells (B) treated with indicated concentrations of gemcitabine for 24 h (black squares) and 48 h (red circles).** IC_50_ values for 48 h treatment are indicated in the text.

Survival rates of two cell lines treated with GEM are shown in Figure 1. The percent survival for both cell lines was less 48 h after treatment compared to 24 h. Inhibition caused by 0.038 μM and 0.38 μM GEM was not statistically significant in either cell line 24 or 48 h after treatment. Since cell death 24 h after treatment had not reached 50%, IC_50_ values were determined at that time. After 48 h IC_50_ values for MCF-7 and MDA-MB-231 exposed to GEM are 13.49 μM and 9.54 μM, respectively. In order to include a concentration of GEM that was below levels of toxicity, 0.38 μM GEM was chosen for all combination treatment with various nsPEF strengths.

### Cytotoxicity induced by combination treatment with NsPEF and gemcitabine

Two strategies for treatments with combinations of nsPEFs and GEM were used. One approach was to treat with a low concentration of GEM to sensitize cells to nsPEF treatment. The other approach was to use nsPEFs to sensitize cells to low GEM treatment. The former approach is analogous to radiosensitization with chemotherapeutic agents sensitizing cells or tumors to radiation treatment.

**Figure 2:**
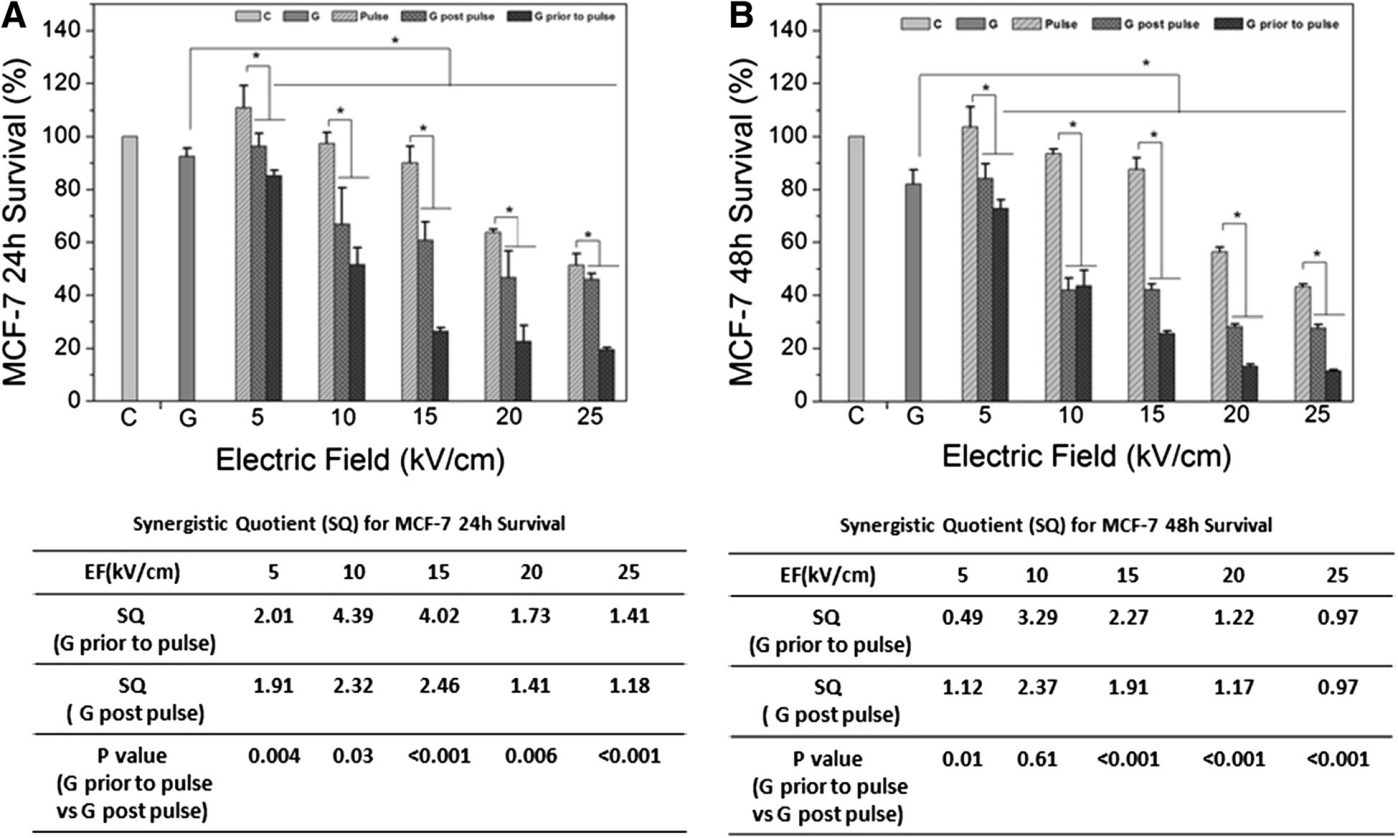
**MCF-7 cell survival after treatment with gemcitabine, nsPEFs and combination – Experiments were carried out with MCF-7 cells as described in Methods for survival 24 h (A) and 48 h (B) after nsPEF treatment.** Synergism quotients (SQ) are shown for both treatment orders. The statistical significance among groups of nsPEFs, GEM and combination treatment was calculated with ANOVA analysis in SPSS. The significance between two groups (two orders of treatments) was calculated with Student-*t* test.

Figure 2 shows survival studies 24 (A) and 48 (B) when MCF-7 cells were treated with GEM before and after treatment with nsPEFs. In order to maximize potentials for synergism, a low concentration of GEM was used with increasing nsPEF treatments with electric fields from 5–25 kV/cm. Tables at the bottom of Figures A and B indicate synergism quotients for corresponding bar graphs above that show specific results of GEM alone, nsPEF alone and combinations with different orders of treatment. The synergism quotient is defined as the ratio of the effect of the combination treatment divided by the sum of the two individual responses. A quotient greater than one indicates that the combination treatment is better. GEM alone caused about a 5% decrease in viability and electric fields alone caused linear decrease in viability from no significant effect at 5 kV/cm to about 50% cell death at 25 kV/cm. As shown by the synergism quotient in Tables below the figure for 24 h (Figure 2A) and the 48 h (Figure 2B) after treatment, regardless of the order of treatments, there was a biphasic effect on synergism, with synergism greater for 10 and 15 kV/cm than at lower or higher electric fields. Another trend was that synergism was greater after 24 h than 48 h after treatments. Finally for both 24 h and 48 h survival experiments, treatments with GEM before nsPEFs were better than GEM treatments after nsPEFs. When cells were first treated with GEM, synergism quotients were from >4 at 24 h and > 3 at 48 h. A similar pattern was seen when cell were treated with GEM after nsPEFs treatment, but the quotients were around 2. Observations of synergism at 24 h and 48 h after treatment indicated that survival mechanisms differed for GEM and nsPEFs in MCF-7 cells.

**Figure 3:**
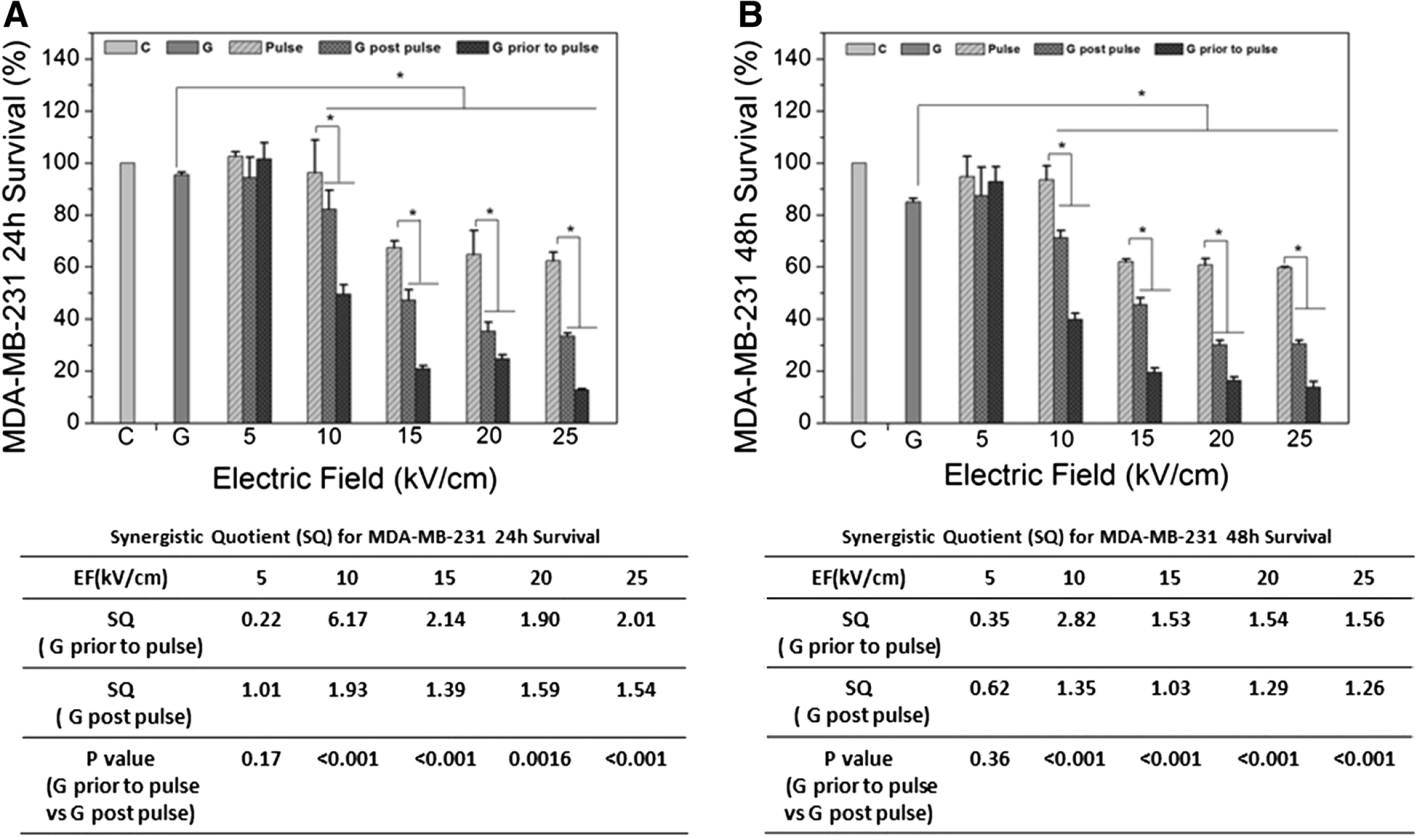
**MDA-MB-231 cell survival after gemcitabine, nsPEFs and combined treatment - Experiments were carried out with MDA-MB-231 cells as described in Methods for survival 24 h (A) and 48 h (B).** Synergism quotients (SQ) are shown for both treatment orders. The statistical significance among groups of nsPEFs, GEM and combination treatment was calculated with ANOVA analysis in SPSS. The significance between two groups (two orders of treatments) was calculated with Student-*t* test.

Figure 3 shows the same survival experiments in the same order for MDA-MB-231 cells as that shown for MCF-7. The 24 h experiments are in Figure 3A and 48 h experiments are in Figure 3B. GEM alone caused about a 5% decrease in viability and electric fields alone caused no significant decrease in viability at 5 kV/cm to about 40% decrease at 25 kV/cm. However, unlike the linear decline in survival, nsPEF effects plateaued between 15 and 20 kV/cm. Synergism was biphasic with greater synergism seen at 10 kV/cm, but synergism tended to be greater at higher electric fields due to the nsPEF plateau at higher electric fields. Nevertheless, synergism was greater at 24 h than 48 h regardless of the treatment order. Like that seen for MCF-7, in MDA-MB-231 cells for both 24 h and 48 h survival experiments, treatments with GEM before nsPEFs were better than GEM after nsPEFs. When cells were treated with GEM first, synergism quotients were from to >6 at 24 h to >2 at 48 h. Observations of synergism at 24 h and 48 h after treatment indicated that survival mechanisms differed for GEM and nsPEFs in MDA-MB-231 cells.

### Effects of nsPEF, gemcitabine and combination treatment on clonogenics in MCF-7 and MDA-MB-231 cells

**Figure 4:**
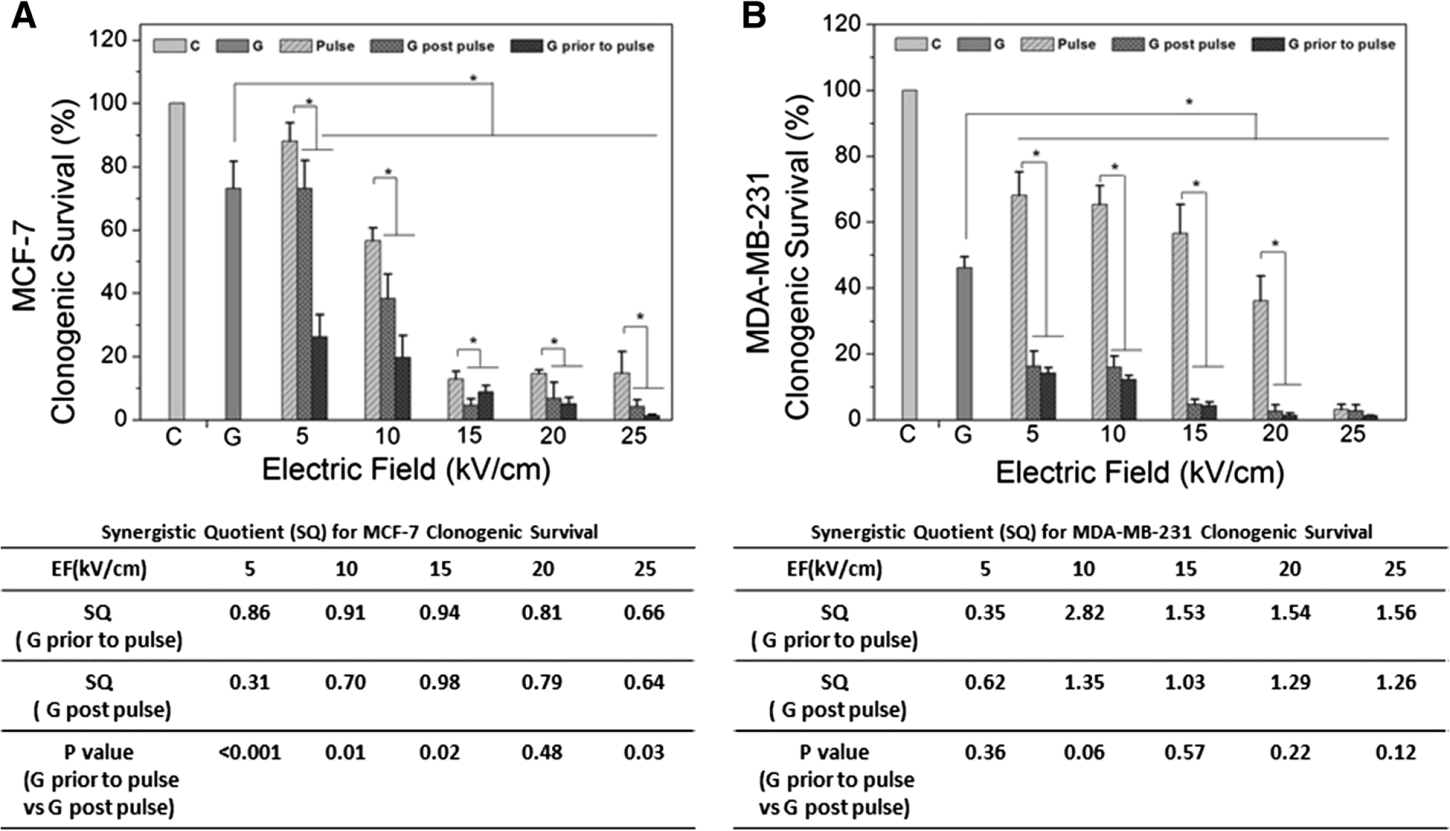
**Clonogenic survival for MCF-7 cell and MDA-MB-231 after gemcitabine, nsPEFs and combined treatment – Clonogenic survival assays and statistics are described in Methods for MCF-7 cells (A) and MDA-MB-231 cells (B).** Synergism quotients (SQ) are shown for both treatment orders.

In order to determine effects of GEM, nsPEFs and combinations of both on populations of cells to undergo unlimited cell division and form colonies, Figure 4 shows clonogenic assays for MCF-7 cells with GEM before and before nsPEFs (panel A) and for MDA-MB-231 cells with GEM before and after nsPEFs (panel B). Clonogenic survival is shown on the y-axis and nsPEFs in kV/cm are shown on the x-axis. The tables under each figure show synergism quotients for the respective treatments in each cell line.

MCF-7 cells did not exhibit synergism at any electric field tested for combinations of GEM and nsPEFs regardless of the order of treatment. In contrast, MDA-MB-231 cells did show synergism between 10 and 20 kV/cm, especially when GEM treatment was prior to nsPEF treatment. Synergism at 25 kV/cm was less meaningful since survival values were so low. Lower synergism might be expected at later times since one or both agents could have optimally activated their respective pathway.

By the end of the clonogenic assay, all cell signaling pathways will have been fully expressed and likely with redundancy; the final results here indicate the end consequences of the treatments. Thus, the aftermath of MCF-7 cell response to GEM and nsPEFs suggests that they share common mechanisms of cell death. In contrast, the after-effect of MDA-MB-231 cell responses suggests that GEM and nsPEFs have different mechanisms for cell death. In this way, GEM and nsPEFs have different effects on these two human mammary cell lines.

### Apoptosis and necrosis mechanisms in response to combination treatments with NsPEFs and gemcitabine

**Figure 5:**
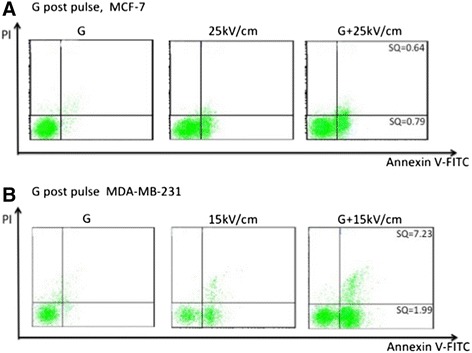
**Typical figures analyzing Annexin-V-FITC binding and propidium iodide (PI) staining for MCF-7 and MDA-MB-231 cells treated with gemcitabine, nsPEFs and combinations.** Experiments were carried out as described in Methods and shown with GEM alone (0.38 μM), nsPEF alone at indicated electric fields and GEM after treatment with nsPEFs. Panel **A** shows an experiment with MCF-7 cells and panel **B** show an experiment with MDA-MB-231 cells. Cell populations are shown in quadrants with Annexin-V-FITC levels indicated on the X-axis and PI staining indicated on the Y-axis. Cells with Annexin-V-FITC labeling only are considered apoptotic (lower right quadrant) and cells with Annexin-V-FITC and PI labeling are considered necrotic (upper right quadrant). Synergism quotients are shown for each cell death type in the respective quadrants. A typical experiment is shown for each cell line.

**Figure 6:**
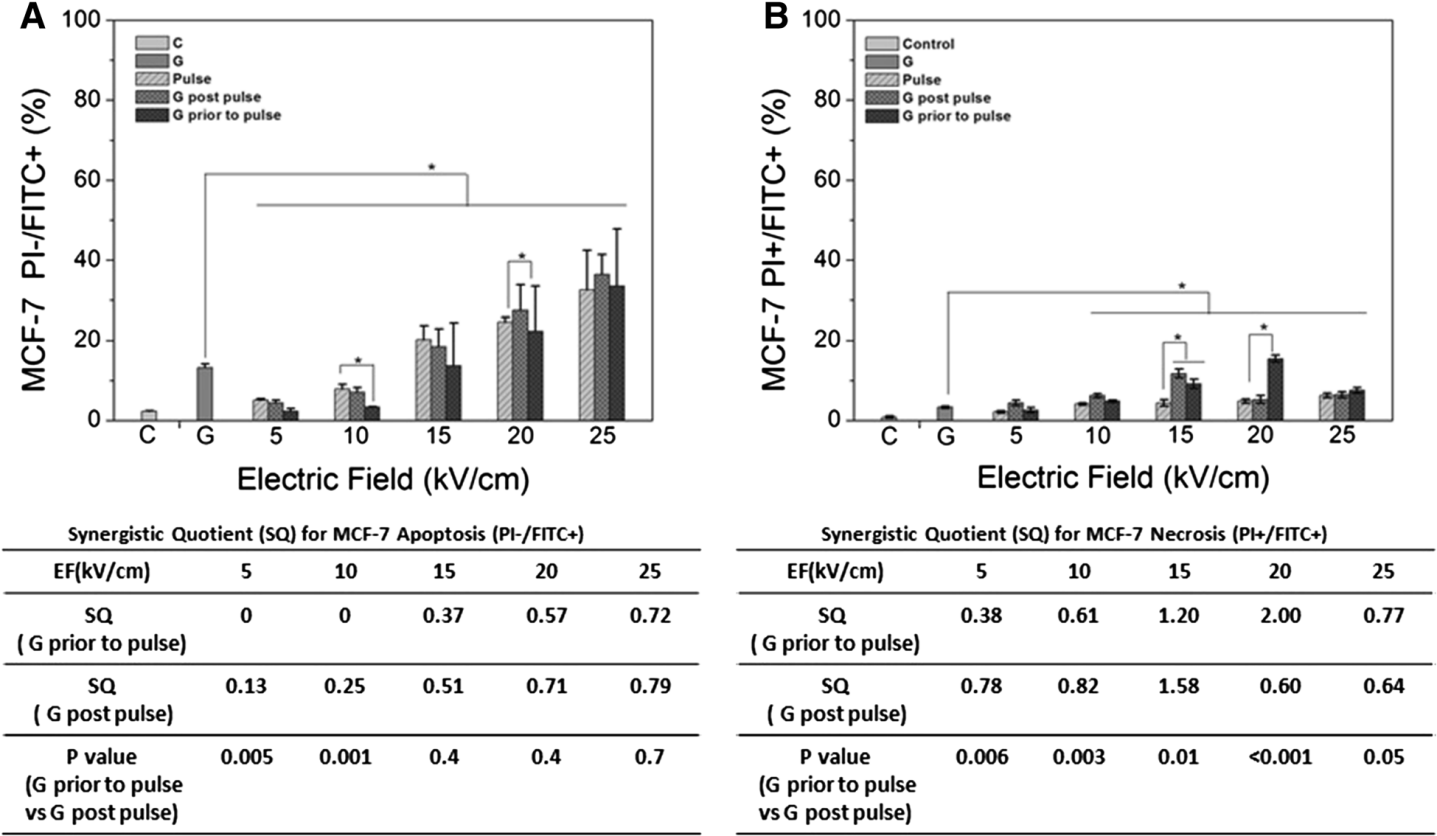
**Cell apoptosis and necrosis for MCF-7 cell after gemcitabine, nsPEF and combined treatment – Experiments were carried out as described in Methods.** Panel **A** shows MCF-7 cell responses for apoptosis (PI-/FITC+) and panel **B** shows MCF-7 cell response for necrosis (PI+/FITC+). GEM was used at 0.38 μM. Data show the apoptotic or necrotic percentages in 10,000 cells analyzed by flow cytometry.

**Figure 7:**
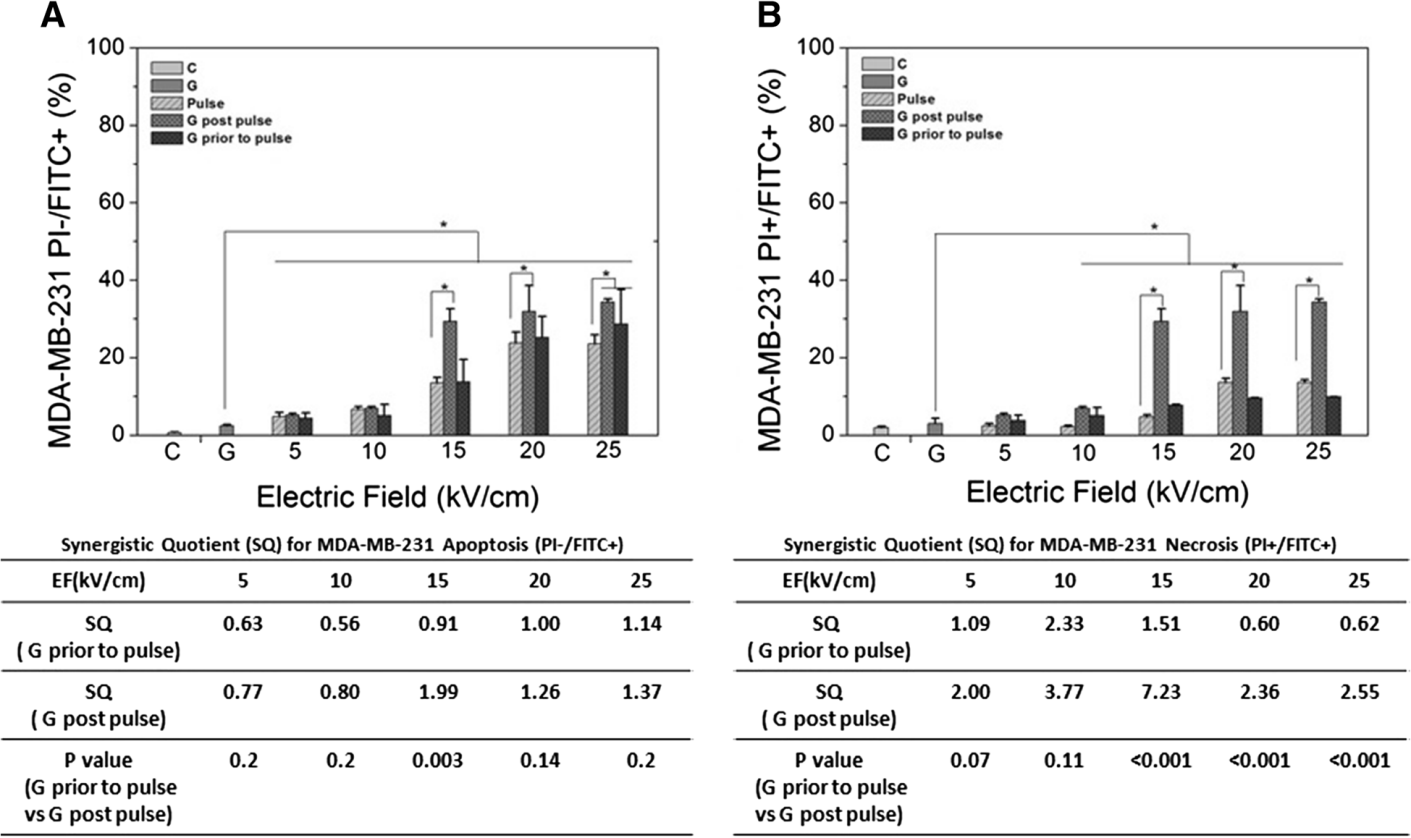
**Cell apoptosis and necrosis for MDA-MB-231 cell after gemcitabine, nsPEF and combined treatment - Experiments were carried out as described in Methods.** Panel **A** shows MDA-MB-231 cell responses for apoptosis (PI-/FITC+) and panel **B** shows MDA-MB-231 cell response for necrosis (PI+/FITC+). GEM was used at 0.38 μM. Data show the apoptotic or necrotic percentages in 10,000 cells analyzed by flow cytometry.

The survival and clonogenics assays suggest that there are differences between cell death mechanisms for MCF-7 and MDA-MB 231 cells. In order to characterize cell death mechanisms into two different categories, Annexin-V-FITC (Annexin) and propidium iodide (PI) assays for apoptosis and necrosis (permeabilized cell membrane) were carried out by flow cytometry. Figure 5 shows a typical experiment with each cell line that was used to generate data in Figures 6 and 7.

Figure 5A shows an experiment with MCF-7 cells that were pulsed with 25 kV/cm with or without GEM added after the pulses. After treatment, MCF-7 cells exhibited little or no Annexin-V positive or PI/Annexin-V positive cells with GEM alone. After the nsPEF treatment, significant numbers of cells became Annexin-V positive (lower right quadrant) and small cell numbers were positive for both markers (upper right quadrant). Still, as indicated by the synergistic quotients less than 1.0, there were no synergistic responses when both agents were present for either cell death condition. Figure 5B shows the same experiment design with MDA-MB-231 cells. These cells exhibited little or no Annexin-V positive or PI/Annexin-V positive cells with GEM alone. In contrast, pulses with an electric field strength of 15 kV/cm produced cells positive for both cell death markers. When both agents were included, there were significant synergistic responses for Annexin-V positive cells only (lower right quadrant; nearly twice as many positive cells as the sum of the two individual responses) as well as synergist responses for cells positive for both markers (upper right quadrant; more than 7 times the response with the combination than the sum of the two individual responses.

Figure 6A shows Annexin+/PI- (apoptosis) and Figure 6B shows Annexin+/PI + (necrosis) for MCF-7 cells 2 hours after treatment with GEM treatment before and after nsPEFs. The tables below each figure indicate synergism for GEM before and after nsPEF treatment. Using these measurements, the occurrence of apoptosis was much greater than necrosis in MCF-7 cells; however, there was no synergism with apoptosis regardless of the order of treatments. The occurrence of necrosis was so low that synergism values were less meaningful. At this 2 h snapshot in MCF-7 cells, GEM and nsPEFs both induce apoptosis through the same or similar pathways without much expression of necrosis.

Figure 7A shows Annexin+/PI- (apoptosis) and Figure 7B shows Annexin+/PI + (necrosis) for MDA-MB-231 cells 2 hours after treatment with GEM before and after nsPEFs. The tables below each figure indicate synergism for GEM before and after nsPEF treatment. In contrast to MCF-7 cells, MDA-MB-231 cells exhibited about equal levels of apoptosis and necrosis. While there was no synergism for apoptosis with either treatment order, there was some synergism for necrosis in MDA-MB 231 cells and in contrast to survival and clonogenics, synergism was slightly stronger when nsPEFs sensitized cells to GEM. At this 2 h snapshot in MDA-MB-231 cells, GEM and nsPEFs both induce apoptosis through the same or similar pathways, but they induce different mechanisms of necrosis.

## Discussion

NsPEFs have been shown to eliminate a number of different tumor types in several different animal models including tumors implanted ectopically, orthotopically or as xenographs. (See [25] for a review). NsPEFs are non-thermal and no safety concerns or side effects have been reported in pre-clinical studies. Effects of nsPEFs have not been determined for breast cancer cells and cancer therapy nearly always includes more than one treatment either sequentially or simultaneously. The strategy here was to look for agents that sensitize to enhance efficacy of a second treatment. Searching for optimal therapeutic sensitizers has been a cancer therapy goal for over 50 years and GEM has been known as a potent sensitizer for 3 decades [16]. A common approach has been to use chemotherapeutic agents to sensitize cancer cells to ionizing radiation. However, other combinations include sensitizing cancer cells with anti-angiogenic factors, EGFR blockade or Cox-2 inhibitors, among others [17]. The strategy used in the present study was to use an agent that is active against a variety of tumors at a low, non-toxic dose so that it sensitizes tumors to another agent, thus resulting in a synergistic response. This has two practical advantages. First, in the present paper both the sensitizing agent and nsPEFs can be used at more modest “doses” meaning low, non-toxic concentrations of GEM and lower electric fields and/or fewer pulses for nsPEFs. Second, because synergism between two agents indicates that they have different mechanisms of action, seeking the presence or absence of synergism for several different GEM and nsPEF responses introduces some new considerations about mechanisms of action for these cancer therapies on two human mammary cell lines. Going a step further, differences in agent-induced cell death and/or survival mechanisms between these two cell types implies that their breast cancer disease mechanisms are very likely different.

Combinations of GEM and nsPEFs resulted in the presence of synergism for 24 h and 48 h survival with both mammary cancer cell types. Therefore, at these indicated times, the overall responses to these agents must have been initiated from different sites and/or propagated through different pathways for survival and/or cell death. For 24–48 h survival, synergism was strongest at earlier times and at intermediate electric fields. Under these conditions, both treatments were acting below their IC_50_ values, perhaps at their IC_15–30_ values so that the “window” or “headroom” for synergism is sufficiently available. When any one treatment approaches its maximum effect, the window or headroom is reduced and synergism cannot be readily observed.

Observations of apoptosis and necrosis 2 h after treatment provided a relatively early snapshot of initial cell responses to GEM, nsPEFs and combination treatments. The GEM-nsPEF-induced cell death mechanisms were different for the two cell types indicating that their cancer disease mechanisms were also likely different. For MCF-7 cells, apoptosis was a major mechanism with very low levels of necrosis. In contrast, MDA-MB-231 cells expressed similar levels of apoptosis and necrosis. Interestingly, there was no synergism for apoptosis in MCF-7 and low synergism levels in MDA-MB-231 cells. Thus, these agents likely use similar mechanisms and/or share common pathways for cell death induction.

Although several GEM-induced mechanisms are likely operative, a major action is to inhibit DNA synthesis by inhibiting ribonucleotide reductase thereby depleting intracellular pools of dCTP/dATP [48], leading to apoptosis through a caspase-8 and mitochondria pathway(s) [49]. However, cell death and apoptosis mechanisms are likely cell- and stimuli-dependent. Likewise for nsPEFs, several cell death mechanisms are likely working, but one defined *in vitro* mechanism is through dissipation of the mitochondrial membrane potential, which is rapid and significant [27]. This is consistent with the observed cytochrome c release from mitochondria [37] and caspase-dependent (apoptosis) (as well as caspase-independent) mechanism(s) through an intrinsic pathway(s) as defined in Jurkat cells *in vitro* [38]. However, there is other indirect evidence that extrinsic apoptosis mechanisms may also be operative [50-52]. Based on the absence of synergism shown here, GEM and nsPEFs are hypothesized to activate apoptosis by similar mechanisms. While specific pathways could be different, it is possible that actions through intrinsic and/or extrinsic pathways that co-activate caspase-3 could function additively in response to these two cell death stimuli. Synergism for apoptosis is not uncommon. For example, it often occurs when a sensitizing agent induces expression of a receptor that is used by the second agent [53].

One possible scenario could be that GEM-induced DNA damage leads to increased expression of Noxa and Puma, which induce cytochrome c release from mitochondria. NsPEF-induced loss of the mitochondria membrane potential could also cause cytochrome c release through a shared pathway with GEM. These mechanisms would not necessarily show synergism for apoptosis.

Major actions of GEM and nsPEFs on MDA-MB-231 were nearly equal for apoptosis and necrosis. However, in contrast to MCF-7 cells, MDA-MB-231 cells exhibited significant synergism for necrosis. Given that these cells are seen as intact entities by flow cytometry, they are not immediately eliminated through an acute cell injury and membrane rupture and are likely undergoing programmed necrosis or necroptosis [54]. The presence of GEM-nsPEF-induced synergism suggests that different necroptotic mechanisms are operative in MDA-MB-231 cells and they can be differentially activated by GEM and nsPEFs. Necroptosis has now been shown to function in physiological processes during development and homeostasis as well as in pathological activities including inflammatory diseases [55]. Necroptosis is relatively well defined through cell death receptors. When ligands bind to death receptors, the cytoplasmic receptor tail recruits multiple proteins that form supramolecular complexes that can initiate cell survival, apoptosis or necroptosis depending on the composition of the complex. While the makeup of these complexes depends on the activated death receptor, phosphorylation of RIP1-RIP3 kinase complexes appears to be important for induction of necroptosis [56]. Although necroptosis pathways in response to GEM or nsPEFs have not been investigated and specific control of RIP1 and/or RIP3 phosphorylation that regulates cell survival remains to be determined, differential regulation of phosphorylation provides potentially powerful sites for synergistic or cooperative regulation of cell signaling.

That GEM can sensitize mammary cancer cells to nsPEF is analogous to radiosensitization with GEM sensitizing tumor cells to radiation [16]. However, the sensitization of cells by GEM and nsPEFs is greater than that observed with radiosensitization. Synergism quotients or enhancement ratios with nsPEFs and GEM here were as high a 6 or 7, while enhancement ratios for radiosensitization were 1.36 to 1.81 [48] and 1.30 to 2.82 [57]. Sensitization of cells with GEM and nsPEFs also differs from radiosensitization, because radiosensitization is not reversible; GEM can sensitize cells to radiation, but radiation cannot sensitize cells to GEM. In contrast, for GEM and nsPEFs, either agent can sensitize the other. However, in general GEM is a better sensitizer for nsPEFs as opposed to nsPEFs sensitizing GEM. GEM-induced inhibition of ribonucleotide reductase, the subsequent depletion of the dATP pools and redistribution of cells into S-phase is required for GEM sensitization to radiation [57,58]. GEM also has some effects on apoptosis; however, the role of apoptosis in radiosensitization depends on the cell line rather than representing a general property of the drug [48]. In other words, it may be due to the cancer disease mechanism(s) in the cell. This is also likely to be the case for GEM sensitization to nsPEFs.

When clonogenics were analyzed for these cells, only MDA-MB-231 showed synergistic responses, indicating that in the final aftermath of treatment, GEM and nsPEFs induced different mechanisms of survival/cell death. It was interesting that MCF-7 cells exhibited synergism 24 h and 48 h after treatment with GEM and nsPEFs, yet did not show synergism with clonogenic nor for apoptosis nor necrosis. This can possibly be explained by a wide range of mechanisms that are activated directly and indirectly by these agents that not only include cell death pathways but also stress, autophagy and cell survival mechanisms. Thus, synergism with MCF-7 cells in 24 h and 48 h survival studies may include mechanisms and pathways associated more with repair and survival than with cell death.

Although GEM and nsPEF combination treatments have not been used in an animal model, there are several different strategies to consider for using these combined treatments *in vivo*. For example, based on these data, animals could be treated first with low, non-toxic concentrations of GEM for various times to sensitize tumors to nsPEFs. GEM treatment could be administered systemically or locally, and could be present or absent when nsPEF treatment occurs. It would also be reasonable to first treat with nsPEFs and then GEM. In any scenario, there are advantages to these strategies that could decrease recurrence and lower incidence of drug resistance. With any of these treatment protocols, cancer cells would not be exposed to long term drug treatment, which can induce different cancers with resistant phenotypes over time; the GEM dose is low and treatment time limited and consequences of a single nsPEF treatment is fast. Although it has not been directly shown, another possible advantage is that high nsPEFs can very likely eliminate cancer stem cells because unlike chemotherapy, nsPEF ablation can eliminate all cells regardless of their proliferation rates when electric fields are sufficiently high [59].

## Conclusions

These studies show enhanced cytotoxicity when GEM and nsPEFs are combined. The treatment order is flexible because either agent can sensitize the other. Analysis of synergism indicates that GEM and nsPEFs share common mechanisms and pathways of cell death, especially in MCF-7 cells; however, the two agents exhibit different mechanisms of necrosis, most likely necroptosis, in MDA-MB-231 cells. These findings also indicate that mechanisms of oncogenesis are different in the two cell lines. Finally, these studies show that combination treatment with GEM and nsPEFs should provide an advantageous therapy for breast cancer ablation *in vivo*.

## Methods

### NsPEF generator

The nsPEF generator was established on a transmission line circuit that was charged from a high-voltage power supply [60]. The breakdown voltage was adjusted by the distance of spark gap. The waveforms were monitored using a digital phosphor oscilloscope (DPO4054. Tektronix. USA) equipped with a high voltage probe (P6015A.Tektronix.USA).

### Cell lines and cell culture

Human breast cancer cell line MCF-7 and MDA-MB-231 were gifts from Prof. Jiangzhong Xi of the Department of Biomedical Engineering in Peking University. Both cell lines were cultured in Dulbecco’s modified Eagle’s medium (DMEM, ATCC formula) with 10% FBS (Sijiqing, Hangzhou, China) and supplemented with 1% penicillin-streptomycin. Cells were maintained in the atmosphere of 5% CO2 at 37°C.

### NsPEF applications

Cells were cultured as monolayers and maintained in exponential growth in a humidified atmosphere with 5% CO_2_/95% at 37°C. When cells were 60-70% confluent they were detach from dishes with 0.25% trypsin (Gibco) and then suspended in complete growth medium. A 400 μl cell suspension (1.2 × 10^6^/ml) was dispensed into cuvettes (Bio-Rad) with 2 mm gaps and exposed to 250 pulses with duration of 65 ns and electric field strengths of 15 kV/cm, 20 kV/cm and 25 kV/cm. The temperature of the cell suspension was recorded every 15 seconds during pulsing. After 250 pulses at 25 kV/cm, the temperature in the cell suspension increased less than 2°C (23.6-25.2°C). The temperature change was within the tolerance of cells, so the measured effects were not due to thermal changes of nsPEF.

### Gemcitabine exposure and combination treatments of gemcitabine and nsPEF

For gemcitabine treatment, cells were detached with trypsin and then dispensed into 96-well plates and incubated with GEM (0.038, 0.38, 3.8, 38.0, and 380.0 μM) for 24 h or 48 h and then assayed with MTT. For nsPEF treatment, cells were detached and dispensed in cuvettes and treated with nsPEFs at electric fields of 0 15, 20 and 25 kV/cm. Cells were then seeded into 96-well plates and cultured for 24 h/48 h before MTT assays. In the treatment of sensitizing cells with nsPEFs before GEM treatment, cells were detached and treated with nsPEFs, then dispensed into 96-well plates and incubated with GEM for 24 h and 48 h before MTT assay. In the treatment of sensitizing cells with GEM before nsPEFs exposure, cells were incubated with 0.38 μM GEM for 24 h before they were detached with trypsin and pulsed. Cells were then seeded into 96 well plates, incubated for 24 h before MTT assays. A synergism quotient is defined as the effect (response) of the combination treatment divided by the sum of the two individual effects (responses). Thus, any value greater than 1.0 indicates synergism. The presence of synergism indicates that the two treatments act at different sites and/or through different mechanisms of action for the measured response. The absence of synergism indicates that the two treatments act at common sites and/or through common pathways.

### Cell viability

Cells were dispensed into 96-well plates and incubated with 20 μl freshly prepared MTT solution (5 mg/ml) at 37°C. For each sample there were 6 replicates. After 4 hours, culture medium was removed and 150 μl DMSO was added to dissolve the purple formazan crystals, and then OD values were obtained by a microplate reader at 492 nm. The survival fraction (SF) was calculated as:

$$ SF=\frac{O{D}_T}{O{D}_C}\times 100\% $$

where OD_C_ and OD_T_ were the optical absorption (OD) values for control and treated groups, respectively.

### Flow cytometry

Was performed according to instructions of the Annexin V-FITC apoptosis kit (Sigma). For individual treatments, 0.38 μM GEM was incubated for 24 h and then analyzed by flow cytometry. Cells were treated with nsPEFs and analyzed 2 h later by flow cytometry. For combination treatments GEM was incubated for 24 h, then treated with nsPEFs and analyzed by flow cytometry 2 h later. Alternatively, cells were treated with nsPEFs and then 0.38 μM GEM was added and incubated for 2 h before analysis by flow cytometry. After treatments, 1 × 10^6^ cells were harvested and washed twice by PBS and resuspended in 500 μl binding buffer. Then 5 μl Annexin-FITC and 5 μl propidium iodide (PI) were added to the suspension and incubated in dark for 15 min before detection by flow cytometry (FACSCalibur). Viable cells are defined as Annexin V-FITC and PI negative; apoptotic cells are defined as Annexin V-FITC positive, PI negative; necrotic cells are defined as Annexin V-FITC and PI positive.

### Clonogenic assay

Cells in log phase growth were collected and exposed to nsPEFs and then seeded into 6-well plates in complete growth medium. For GEM treatment or the combination treatment, medium with 0.38 μM GEM was then added to cells. Cells were incubated at 37°C for 7 days. After washing twice with PBS, cells were fixed with 4% glutaraldehyde for 30 min and then dyed with Giemsa solution. Cell colonies with greater than 50 cells were counted with microscope. Plating efficiency (PE) and cell survival $SF=\frac{N{P}_T}{N{C}_T\times PE}\times 100\%$ fraction (SF) were calculated as following:

$$ PE=\frac{N{P}_C}{N{C}_C}\times 100\% $$

where NP_C_ was the number of colonies counted and NC_C_ was the number cells seeded in the control group, while NP_T_ and NC_T_ were the number of colonies counted and cells seeded, respectively, in the treated group.

### Statistical analysis

All data were processed by Origin Professional 8.0 software. The statistical significance among groups of nsPEF, GEM and combination treatment was calculated with ANOVA analysis in SPSS. The significance between two groups (two orders of treatments) was calculated with Student-*t* test) and considered when p < 0.05.

## Abbreviations

GEM: gemcitabine
nsPEFs: nanosecond pulsed electric fields
DCIS: Incidences of ductal carcinoma *in situ*
HCC: hepatocellular carcinoma
Annexin: Annexin-V-FITC
PI: propidium iodide
apoptosis: PI-/Annexin+
necrosis: PI+/Annexin+
